# Cemented vs Uncemented hemiarthroplasties for femoral neck fractures: An overlapping systematic review and evidence appraisal

**DOI:** 10.1371/journal.pone.0281090

**Published:** 2023-02-24

**Authors:** Arjun K. Reddy, Jared T. Scott, Grayson R. Norris, Chip Moore, Jake X. Checketts, Griffin K. Hughes, Travis Small, Mark M. Calder, Brent L. Norris

**Affiliations:** 1 Department of Orthopaedic Surgery, Oklahoma State University Medical Center, Tulsa, Oklahoma; 2 Office of Medical Student Research, Oklahoma State University Center for Health Sciences, Tulsa, Oklahoma; 3 Orthopaedic & Trauma Service of Oklahoma, Tulsa, Oklahoma; 4 Department of Orthopaedic Trauma, The University of Oklahoma at Tulsa School of Community Medicine, Tulsa, Oklahoma; Baqai Medical University, PAKISTAN

## Abstract

**Background:**

The purpose of our study is to assess the methodology of overlapping systematic reviews related to cemented vs uncemented hip hemiarthroplasties for the treatment of femoral neck fractures to find the study with the best evidence. Also, we assess the gaps in methodology and information to help with direction of future studies.

**Methods:**

A systematic search was conducted in September 2022 using Pubmed, Embase, and Cochrane Library. Clinical outcome data and characteristics of each study were extracted to see which treatment had better favorability. The outcomes and characteristics extracted from each study includes, first author, search date, publication journal and date, number of studies included, databases, level of evidence, software used, subgroup analyses that were conducted, and heterogeneity with the use of I^2^ statistics Methodological quality information was extracted from each study using four different methodologic scores (Oxford Levels of Evidence; Assessment of Multiple Systematic Reviews (AMSTAR); Quality of reporting of meta-analyses (QUROM); Oxman and Guyatt. After that, the Jadad decision algorithm was used to identify which studies in our sample contained the best available evidence. Finally, overlap of each systematic review was assessed using Corrected Covered Area (CCA) to look at redundancy and research waste among the systematic reviews published on the topic.

**Results:**

After screening, 12 studies were included in our sample. For the Oxford Levels of Evidence, we found that all the studies were Level I evidence. For the QUORUM assessment, we had 1 study with the highest score of 18. Additionally, we did the Oxman and Guyatt assessment, where we found 4 studies with a maximum score of 6. Finally, we did an AMSTAR assessment and found 2 studies with a score of 9. After conducting the methodological scores; the authors determined that Li. L et al 2021 had the highest quality. In addition, it was found that the CCA found among the primary studies in each systematic review calculated to .22. Any CCA above .15 is considered “very high overlap”.

**Conclusions:**

The best available evidence suggests that Cemented HAs are better at preventing Prosthesis-related complications. Conversely, the best evidence also suggests that Cemented HA also results in longer operative time and increased intraoperative blood loss. When conducting future systematic reviews related to the topic, we ask that authors restrict conducting another systematic review until new evidence emerges so as not to confuse the clinical decision-making of physicians.

## Introduction

As of 2019, the global incidence of hip fractures exceeds 14 million–a 90% increase since 1990 [1]. People who experience hip fractures are prone to a wide range of complications that can result in death and decreased quality of life (QoL) [2–4]. Hip fractures are commonplace within elderly populations; in the United States, over 300,000 older individuals are hospitalized for hip fractures every year [5]. Femoral neck fractures commonly occur in this population, sustained through low-energy falls. For these elderly patients (65 years or older), both total hip arthroplasty and hemiarthroplasty are equally valid treatment options [6–9].

The hemiarthroplasty procedure varies on the nature of the fracture. Surgeons may approach fractures from different angles and planes, consider unipolar vs bipolar femoral head implants, and elect for cement vs non-cemented techniques. Germane to cement and non-cement technique, best practice remains uncertain due to the variation in results from primary studies and meta-analyses. In September 2014, Middleton et al. reported significantly higher re-operation rates for those undergoing an uncemented operation (p = 0.005) [10]. Four months later, Grammatopoulos et al. reported no significant difference in the re-operation rate between the cemented and uncemented groups (p = 0.36) [11]. A meta-analysis conducted by Li et al., as published in *Plos One* reported no significant difference in postoperative hip function between cemented and uncemented hemiarthroplasties at two months (p = .82) [12]. In contrast, Lin et al. published a meta-analysis in *Medicine* that stated that cemented hemiarthroplasties resulted in better postoperative hip function at 12 months (p = .01) [13]. Further, these meta-analyses cite variable outcome measurement and heterogeneity as key barriers to comprehensive pooled analyses, leaving a small sample of trials from which to draw important clinical conclusions.

We believe that the prevalence of small sample sizes, inconsistent outcome measures, and variable heterogeneity may be driving discrepant results in the hemiarthroplasty literature base. The purpose of our study is to conduct an overlapping analysis of systematic reviews/meta-analyses exploring cemented vs uncemented hemiarthroplasty outcomes. Secondly, we plan on conducting methodology assessments among the studies within our sample. Finally, we hope to precisely demonstrate gaps and provide practical solutions for improving the hemiarthroplasty evidence base for future research and improved patient care.

## Materials and methods

This systematic review was done in adherence with the Preferred Reporting Items for Systematic Reviews and Meta-Analysis (PRISMA) statement [14]. Ethical approval or patient consent is not required for this review due to its secondary nature. Our methodology follows a similar design as Zhao et al [15]. A systematic search was conducted in September 2022 using Pubmed, Embase, and Cochrane Library. Two reviewers reviewed each title and abstract followed by a full-text screen to identify articles that met the inclusion criteria. Senior authors settled disagreements. The search string used is as follows: (cement OR cemented) AND (uncement OR uncemented OR noncement OR noncemented OR cementless) AND (hemiarthroplasty OR arthroplasty OR replacement) AND (hip OR femur OR femoral) AND (fracture OR fractures) AND (systematic review OR meta analyses OR meta analysis OR review) (’cement’ OR ’cemented’) AND (’uncement’ OR ’uncemented’ OR ’noncement’ OR ’noncemented’ OR ’cementless’) AND (’hemiarthroplasty’ OR ’arthroplasty’ OR ’replacement’) AND (’hip’ OR ’femur’ OR ’femoral’) AND (’fracture’ OR ’fractures’) AND (’systematic review’ OR ’meta analyses’ OR ’meta analysis’ OR ’review’).

### Inclusion and exclusion criteria

Inclusion criteria for the study were as listed: 1.) Systematic review and Meta-analysis that juxtapose Cemented vs Uncemented Hemiarthroplasties for the treatment of Femoral Neck Fractures; 2.) included sample must contain solely randomized controlled trials (RCTs); 3.) report at least one outcome in comparison of the two treatment modalities; 4.) English-written articles; 5.) Analyses must only include human studies. Exclusion criteria included any systematic review without a meta-analysis or data pooling; also, narrative reviews, meeting abstracts, and meta-analyses with non-RCT studies were excluded.

### Data extraction

For each study included in the sample, we extracted: First author, search date, publication journal and date, number of studies included, databases, level of evidence, software used, subgroup analyses that were conducted, and heterogeneity with the use of I^2^ statistics. Also, clinical outcome data were extracted to see which treatment had better favorability.

### Quality assessment

Methodological information was extracted using four different methodologic scores. The following methodologic scores were conducted for the 11 studies in our sample in a double and blind fashion: Oxford Levels of Evidence [16, 17]; Assessment of Multiple Systematic Reviews (AMSTAR) [18, 19]; Quality of reporting of meta-analyses (QUROM) [20]; Oxman and Guyatt Index [21]. Any disagreements were solved in consensus.

### Heterogeneity assessment

When conducting a meta-analysis, heterogeneity is assessed using I^2^ statistics. When the assessment yields a result that is greater than 50%, then heterogeneity is deemed to exist. Therefore two authors assessed whether sensitivity or subgroup analyses were carried out to identify the source of the heterogeneity.

### Application of Jadad decision algorithm

The Jadad decision algorithm was used to identify which studies in our sample contained the best available evidence. Jadad et al. [22] describe that discordance of meta-analyses is derived from 6 separate reasons: clinical question, study selection and inclusion, data extraction, assessment of study quality, assessment of the ability to combine studies, and statistics used for data synthesis. Two reviewers conducted the algorithm independently, then, through consensus, decided which meta-analysis represents the best evidence.

### Assessment of overlap among primary studies

Two investigators extracted the primary studies from the systematic reviews (SRs) within our sample. Afterward, the overlap of primary studies between systematic reviews was calculated using the corrected covered area (CCA) tool developed by Pieper et al. [23]. Each of the studies will be compared against one another and yield a CCA value. After obtaining all the CCA values between the studies, they will be averaged to get an overall CCA value. The interpretation of CCA is as follows: 0-.05 = slight overlap, .06-.1 = moderate overlap, .11-.15 = high overlap, and >.15 = very high overlap.

## Results

### Literature search

**Fig 1:**
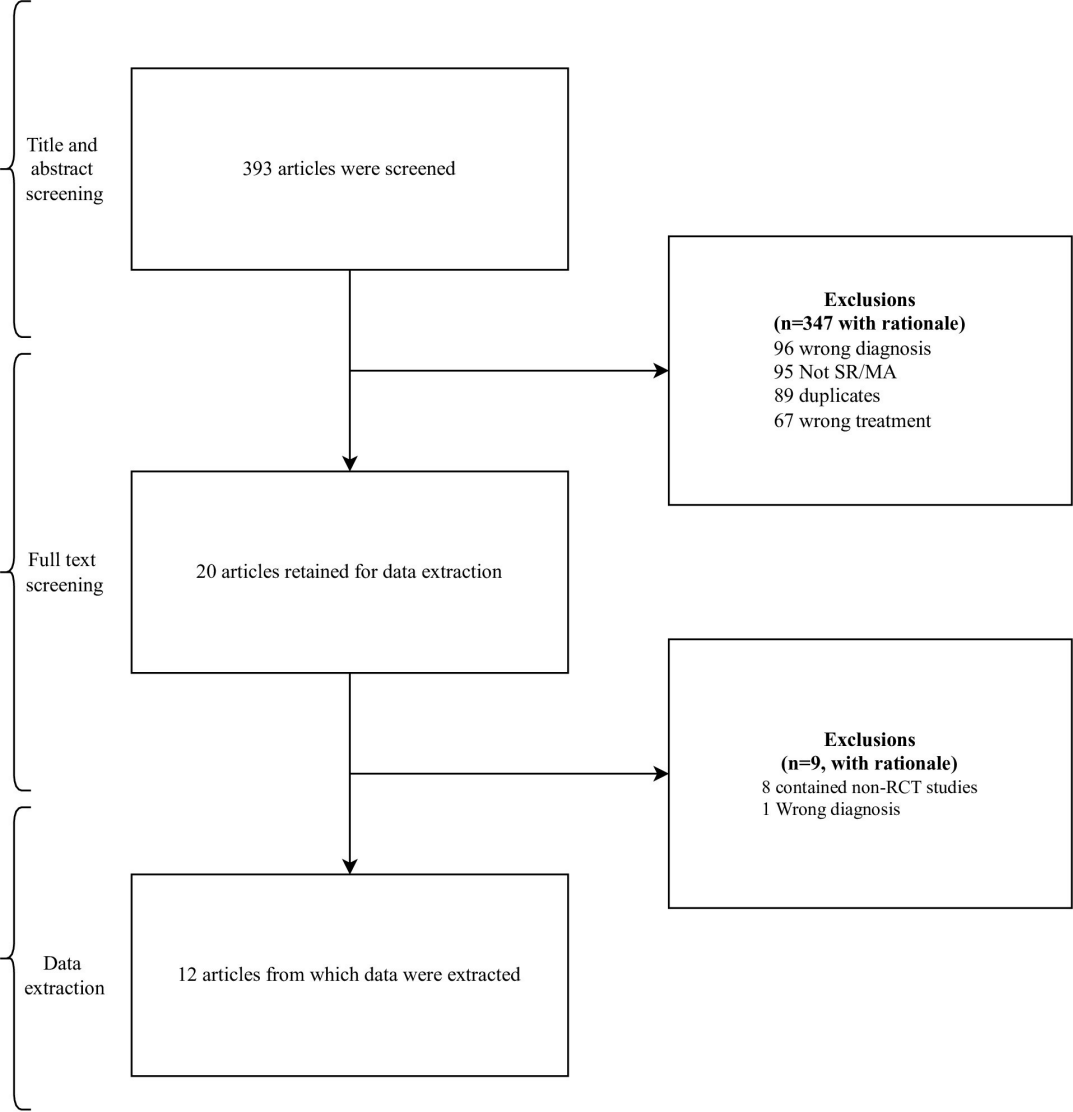
Flow chart.

The initial search string found 393 studies. After screening, 12 studies were included in our sample (Fig 1) [12, 13, 24–33]. The overlapping meta-analyses were published between 2011 to 2021 in different journals, with the number of RCTs in the sample ranging from 5 to 18 (Table 1). The RCTs were published from 1977 to 2021 (Table 2).

**Table 1:** Characteristics.

Author (year)	Publication date	Journal	Search Date	# of RCTs
Liu B (2020)	August 2020	Medicine	December 2018	15
Kong (2020)	June 2020	Experimental and Therapeutic Medicine	February 2019	16
Li N (2020)	February 2020	Medicine	December 2018	8
Luo X (2012)	December 2011	Archives of Orthopaedic and Trauma Surgery	December 2010	8
Kumar P (2019)	January 2019	European Journal of Orthopaedic Surgery & Traumatology	February 2018	4
Azegami S (2011)	September 2011	HIP International	September 2009	8
Li T (2013)	July 2013	PLOS One	December 2012	7
Ning G (2014)	December 2012	European Journal of Orthopaedic Surgery & Traumatology	March 2012	12
Lin FF (2019)	February 2019	Medicine	February 2016	7
Veldman HD (2017)	April 2017	The Bone & Joint Journal	April 2016	5
Nantha Kumar N *(2020)	August 2020	The Bone & Joint Journal	February 2020	18
Li L (2021)	June 2021	Archives of Orthopaedic and Trauma Surgery	January 2020	11

**Table 2:** Included RCTs in each systematic review.

	Liu	Kong	Li N	Luo	Kumar	Azegami	Li T	Ning	Lin	Veldman	Nantha Kumar	Li L
Sonne-Holm 1982 [57]	Y	Y		Y		Y	Y	Y			Y	
Dorr 1986 [58]	Y					Y		Y			Y	
Emery et al. 1991 [59]	Y	Y	Y	Y		Y	Y	Y			Y	Y
Figved 2009 [60]		Y		Y	Y	Y	Y	Y	Y		Y	
Moroni 2009 [61]								Y	Y		Y	
DeAngelis 2012 [62]	Y	Y			Y		Y	Y	Y	Y	Y	Y
Taylor 2012 [63]	Y	Y			Y		Y	Y	Y	Y	Y	Y
Talsnes 2013 [64]	Y	Y	Y						Y	Y	Y	Y
Langslet 2014 [65]	Y	Y			Y				Y	Y	Y	Y
Inngul 2015 [66]		Y							Y		Y	
Harper and Gregg 1992 [67]				Y		Y		Y			Y	
Branfoot 2000 [68]				Y		Y		Y				
Santini 2005 [69]	Y			Y		Y		Y		Y	Y	
Parker 2010 [70]	Y		Y	Y		Y	Y	Y			Y	Y
Cumming and Parker 2012 [71]								Y			Y	
Sadr and Arden 1977 [72]	Y			Y			Y					Y
Vidovic 2013 [73]	Y										Y	
Barenius 2018 [74]	Y										Y	
Moerman 2017 [75]	Y	Y			Y						Y	
Parker and Cawley 2020 [76]		Y									Y	
Vidovic 2015 [77]		Y										
Khorami 2016 [78]	Y	Y										
Li 2017 [79]		Y										
Ma 2016 [80]		Y										
Prashanth and Niranjan 2017 [81]	Y											
Mohabey 2017 [82]					Y							
Movrin 2020 [83]												Y
Du 2014 [84]		Y										
Pan 2013 [85]		Y										

### Search methods for systematic reviews/meta-analyses

While all literature searches were conducted appropriately, we found variations in the databases that were searched. All 12 studies searched the Embase database, while 11 studies searched Cochrane Library, 9 studies searched Pubmed, and 6 studies searched Medline. In addition, databases such as SCOPUS, Web of Science, CNKI, WANFANG, CINAHL, VIP database, and Google Scholar were searched in the included studies. Other aspects of methodology were shown to vary, including language restrictions and publication status restrictions. We found that 5 studies had English-only language restrictions, while 7 studies did not have language restrictions. We also had only 1 study disclose a grey literature search for unpublished literature. Of the other studies, 7 specified that published literature was the only type that would be reviewed by the authors, while 4 studies did not specify. For further information on the search methodology of each study, refer to Table 3.

**Table 3:** Search methodology.

Author (year)	Language Restriction	Publication status Restriction	Pubmed	Embase	SCOPUS	Medline	Cochrane Library	Web of Science	CNKI	WANFANG	CINAHL	VIP Database	Google Scholar
Liu B (2020)	Yes	NA		*		*	*	*					
Kong X (2020)	No	NA	*	*			*		*	*			
Li N (2020)	No	Yes	*	*			*						
Luo X (2012)	Yes	Yes		*		*	*						
Kumar P (2019	Yes	Yes	*	*	*								
Azegami S (2011)	No	No		*		*	*				*		
Li T (2013)	Yes	Yes	*	*			*		*			*	
Ning G (2014)	Yes	Yes	*	*		*	*						*
Lin FF (2019)	No	NA	*	*			*						*
Veldman HD (2017)	No	NA	*	*			*	*					
Nantha Kumar N (2020)	No	Yes	*	*		*	*	*			*		
Li L (2021)	No	Yes	*	*		*	*						

### Methodological quality

There was continued discordance with the different software used by the studies in our sample. The software used in our sample includes RevMan (which 9 studies used), STATA (which 2 studies used), and one study did not specify. All of the studies contained a Sensitivity/Subgroup Analysis. In addition, only 4 of the studies in the sample assessed publication bias. (Table 4).

**Table 4:** Methodologic information.

	Primary Study Design	Level of Evidence	Software use	Grade Use	Sensitivity/Subgroup Analysis	Publication Bias
Liu B (2020)	RCT	I	RevMan	No	Yes	No
Kong X (2020)	RCT	I	RevMan	No	No	Yes
Li N (2020)	RCT	I	RevMan	No	Yes	No
Luo X (2012)	RCT	I	RevMan	No	Yes	No
Kumar P (2019)	RCT	I	RevMan	Yes	Yes	No
Azegami S (2011)	RCT	I	NA	No	No	No
Li T (2013)	RCT	I	RevMan	Yes	Yes	No
Ning G (2014)	RCT	I	STATA	No	Yes	Yes
Lin FF (2019)	RCT	I	RevMan	No	Yes	No
Veldman HD (2017)	RCT	I	RevMan	No	No	Yes
Nantha Kumar N (2020)	RCT	I	STATA	No	Yes	Yes
Li L (2021)	RCT	I	RevMan	Yes	Yes	Yes

We also conducted 4 different methodological quality assessments of the sample to determine the highest quality study. We found that all the studies were Level I evidence based on the Oxford Levels of Evidence. For the QUORUM assessment, we had 1 study with the highest score of 18. Additionally, we did the Oxman and Guyatt assessment, where we found 4 studies with a maximum score of 6. Finally, we did an AMSTAR assessment and found 2 studies with a score of 9. (Table 5) After a consensus discussion, the authors determined that Li. L et al. [33] had the highest quality.

**Table 5:** Methodologic scores.

		QUORUM	Oxman-Guyatt	AMSTAR
Cemented versus uncemented hemiarthroplasty for elderly patients with displaced fracture of the femoral neck	Liu	16	5	7
Meta‐analysis of the effect of cemented and uncemented hemiarthroplasty on displaced femoral neck fracture in the elderly	Kong	16	4	7
Cemented versus uncemented hemi-arthroplasty for femoral neck fractures in elderly patients: A systematic review and meta-analysis of randomized controlled trials	Li	17	5	8
Systematic review of cemented versus uncemented hemiarthroplasty for displaced femoral neck fractures in older patients	Luo	12	3	4
Hemiarthroplasty for neck of femur fractures: to cement or not? A systematic review of literature and meta‐analysis	Kumar	16	6	7
Cemented versus uncemented hemiarthroplasty for hip fractures: a systematic review of randomised controlled trials	Azegami	12	4	6
Cemented versus Uncemented Hemiarthroplasty for Femoral Neck Fractures in Elderly Patients: A Meta- Analysis	Li	17	5	7
Cemented versus uncemented hemiarthroplasty for displaced femoral neck fractures: an updated meta-analysis	Ning	17	4	8
Cemented versus uncemented hemiarthroplasty for displaced femoral neck fractures A meta-analysis of randomized controlled trails	Lin	17	6	7
Cemented versus cementless hemiarthroplasty for a displaced fracture of the femoral neck A SYSTEMATIC REVIEW AND META-ANALYSIS OF CURRENT GENERATION HIP STEMS	Veldman	17	6	8
Effectiveness and safety of cemented and uncemented hemiarthroplasty in the treatment of intracapsular hip fractures A SYSTEMATIC REVIEW AND META-ANALYSIS OF RANDOMIZED CONTROLLED TRIALS	Nantha Kumar	17	5	9
Cemented versus uncemented hemiarthroplasty for the management of femoral neck fractures in the elderly: a meta-analysis and systematic review	Li L	18	6	9

### Heterogeneity evaluation

All of the studies in the sample contained I^2^ statistics. There were varying results in our sample. Certain variables with little to no heterogeneity were cardiovascular complications and reoperations/revisions. Other stats with a high rate of heterogeneity were prosthetic-related complications and operation time. To account for the heterogeneity, 9 studies conducted sensitivity/subgroup analyses. (Table 6).

**Table 6:** Pooled analysis of each meta-analysis and I2 statistics.

	Liu	Kong	Li N	Luo	Kumar	Azegami	Li T	Ning	Lin	Veldman	Nantha Kumar	Li L
HHS/functional	N/51%		N/78%		C/0%		C/0%		N/51% / C/0%		N	N/85%
Postoperative Pain	C/66%			C/0%	N/65%	N/0%	C/0%	N/80.2%				C/0%
Reoperation and Revision	N/0%		N/0%	N/0%	N/0%		N/0%		N/0%	N/0%	N/74% / N/74%	C/0%
General Complications	N/0%			N/0%	C/90%		N/0%		N/0%	N/0%	N	N
Local Complications	N/0%								C/73.3%	N/0%		N
Prosthetic-related Complications	C/41%				C/55%		C/42%			C/41%		C/38%
Intraoperative Fractures				N/0%	N/0%		N/0%		C/0%		C/74%	
Periprosthetic Fractures		C/16%	C/30%						C/0%		C/74%	
Prosthesis Dislocation		N/0%	N/0%						N/0%		N/74%	
Postoperative Deep Infection											N/74%	
Wound Infections									N/7%		N	
UTI/medical		N/0%							N/0%			
Perioperative Mortality							N/0%		N/0%			
0–6 months- mortality		N/0%									N	N/0%
1 year- mortality	N/0%	N/0%	N/0%	N/0%	L/0%	N/0%	N/34%		N/50%	N/0%	N/13%	
Length of Hospital Stay	N/0%	C/84%	N/0%					N/0%		N/0%		
Operation Time	L/45%	L/77%	N/51%		L/64%		L/20%	L/10.9%	L/52%	L/46%	L	L/38%
Intraoperative Blood Loss	N/56%	N/85%	N/57%		N/52%		N/14%	N/36.2%	N/0%	L/41%	N	
Incision Infection		N/0%										
Pulmonary Embolism		N/0%	L/0%									
Cardiovascular	N/0%	N/0%	N/0%				N/0%		N/0%	N/0%		
DVT		N/0%	N/0%									
Pneumonia		N/0%	N/62%						N/0%			
Cerebrovascular incident		N/0%					N/0%		N/0%			
	"Local" and "general" complicationsnot reported in detail			"Local complications"			Cardiovascular and Cerebrovascular complications were merged to gether	- Mortality was not specific on time range -Complications not specified	Cardiovascular and Cerebrovascular complications were merged to gether			

* N = No Difference, C = Cemented HA favored, L = Cementless HA favored

### Results of Jadad decision algorithm

When conducting the Jadad decision algorithm, the methodology helps identify the best current evidence in treatment. The first step is identifying if studies are asking the same question, which they are. While they are asking the same question, they do not have the same number of primary trials in their sample and their criteria for study selection was different. As a result, we conducted 4 different methodological analyses for each of the studies. We found that Li L et al. [33] was the highest methodological study and therefore was selected.

#### Overlap assessment using CCA

**Fig 2:**
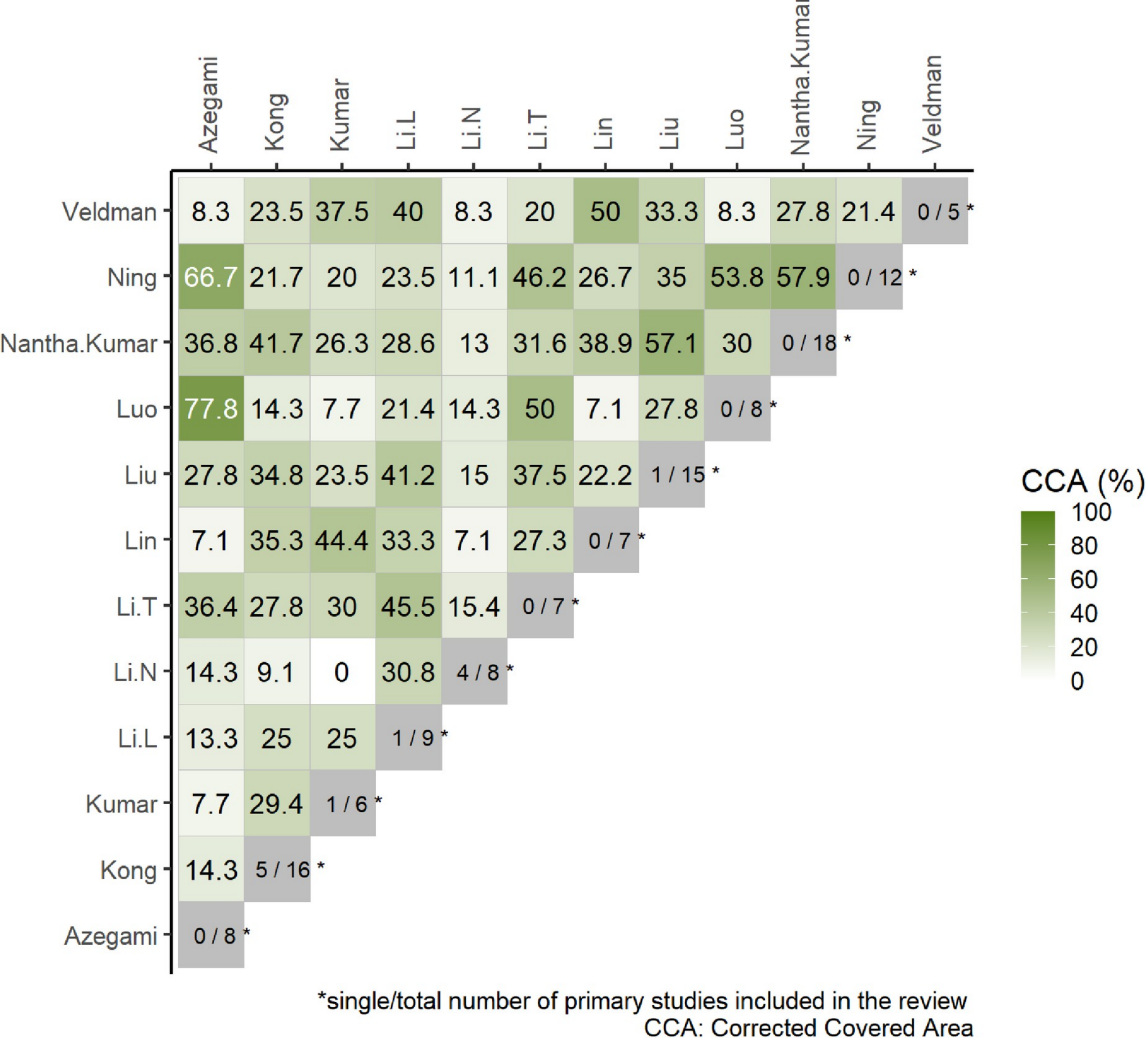
CCA heat map.

We found 34 RCT primary studies cited within the 12 SRs in our sample. After comparing the 12 SRs, our sample yielded 66 CCA data points for each comparison. When gauging each individual comparison, we found that 48 of the 66 comparisons had a CCA value greater than .15. There were 8 CCAs between .10 and .15., 9 CCAs between .06 and .10, and 1 CCA between 0 and .05. After conducting our analysis, our sample contained a CCA value of .22. (Fig 2).

## Discussion

Our study found that the highest quality meta-analyses related to the treatment of femoral neck fractures using cemented vs uncemented hemiarthroplasties (HAs) were Li L et al. [33] Li L et al. found a statistical significance that Cemented had better results in terms of postoperative pain, reoperations and revisions, and Prosthetic related complications compared to Uncemented HAs. On the other hand, Uncemented Arthroplasties were associated with lower operation times compared to Cemented.

Many RCTs have been conducted comparing Cemented and Uncemented HAs as a treatment option for Femoral Neck fractures, showing discordant results. Therefore, multiple meta-analyses were also conducted to pool the data together and see the results. But, these meta-analyses also show discordant results. As a result, there is continued debate on the best treatment method. Overall consensus suggests that the decision should be based on surgeon preference, philosophy, and expertise [34]. Specific attributes of each treatment that physicians consider when deciding which option to use would include Mortality and/or short-term outcomes such as complications, and revisions [35].

### Mortality

In terms of mortality, hip fractures have a high mortality rate that continues to increase [3, 36, 37]. Therefore, treatment must be done promptly. Huette et al. discuss how specific factors such as Age, Time of Surgery, and a Lee Score greater than 3 lead to higher 1-year mortality rates [38]. When considering the type of HA to treat a patient with a hip fracture, physicians are finding disagreement based on the available evidence [39, 40]. We found that of the 10 studies that analyzed the data on 1-year Mortality, 9 studies stated that there was no statistical difference between mortality of Cemented vs Uncemented HA. Kumar et al. was the only study statistically significant for mortality favoring Uncemented HAs [28]. Although there was a statistical difference between the included RCTs, the authors state that the data could have been subject to bias based on intraoperative plan changes based on bone quality and size of the medullary canal.

Reoperation and revision rates are essential to determining treatment since it has been shown that revisions are associated with higher mortality in individuals that undergo hip arthroplasties [41]. Our sample had 8 studies that looked at this, and all studies found that there was no difference in reoperation/revision rates between Cemented and Uncemented HAs. This is intriguing because our sample shows statistical significance for Cemented HAs having a lower prosthetic-related complications rate than Uncemented HAs.

### Prosthetic-related complications

It is agreed upon that patients’ short-term experience after HA surgery is essential for positive outcomes [35]. Similarly to mortality, various studies show discordance regarding short-term outcomes after HA. For example, studies have reported that there is a high risk of thigh pain for the Uncemented implants, in contrast, Rolfson et al. have reported that 1st-year postoperative outcomes are better in patients that undergo Uncemented HA compared to Cemented HA [42–44]. A variety of outcomes were assessed in the meta-analyses within our sample. To start, 4 studies looked at overarching prosthetic-related complications, and all found that Cemented HAs were preferable, with two of the studies showing low heterogeneity associated with their results [12, 24, 28, 31]. We also looked at specific prosthetic-related complications. No statistical significance was found favoring either treatment for avoiding dislocations, but the same studies found statistical significance for Cemented HAs more favorable in avoiding periprosthetic fractures [13, 25, 26, 32].

### Research wastage

Our study also analyzed the overlap in primary literature displayed among the SRs using CCA. It was found that the CCA found among the SRs was .22, which is considered among the highest degree of overlap according to Pieper et al. [23]. In addition to conducting our analysis, we interestingly found, of the 11 studies within our sample, 6 systematic reviews were published on the same topic between 2019–2020 [13, 24–26, 28, 32]. While it is ok for overlap of meta-analyses, there needs to be significant new empirical evidence; however, the publication of numerous meta-analyses over the same topic in a short time frame is concerning, as it could contribute to the confusion already associated with the treatment path of femoral neck fractures [45, 46]. Siontis et al. discuss how the overlap of systematic reviews can be warranted, but the publication of numerous similar meta-analyses over a short period contributes to wasted research efforts and confusion among readers [46]. Authors must be wiery of this and wait to publish more reviews on the topic until higher-quality RCTs are published. In addition, to avoid the redundancy of hemiarthroplasty meta-analyses and improve future studies, we discuss the steps below that can be conducted to improve upon the studies in our sample.

Firstly, we recommend that before authors conduct an SR, they conduct a literature search for existing studies to prevent redundancy in the literature. We also recommend that journals conduct the same literature search during the submission process to see if there is a gap in the literature the study fills. Secondly, only 1 of the studies in our sample looked at unpublished literature when conducting their search [29]. Searches for unpublished literature data are essential, due to the presence of publication bias in orthopaedic literature [47]. Due to publication bias, Cochrane Collaboration requires that systematic reviews published in their journal have at least one search for unpublished clinical trials. It was found that only 7% of Orthopaedic Systematic Reviews conducted a clinical trial registry search with almost 60% of Orthopaedic Systematic Reviews potentially adding unpublished clinical trial data to their sample, allowing for more accurate effect sizes by reducing publication bias [48–50]. In addition, it is necessary for future systematic reviews to conduct assessments on publication bias within their sample. Only 4 of the studies in the sample conducted funnel plots to look for publication bias within their study [25, 30–32]. This is problematic, considering PRISMA states that an assessment of the risk of publication bias should be conducted [51]. This is due to the overrepresentation of significant results in published literature. As a result, underpowered studies can have distorted conclusions due to publication bias [52]. Finally, when conducting meta-analyses, after conducting a sensitivity analysis to identify the heterogeneity within a meta-analysis, it should be common practice to conduct a random-effects model to remove the heterogeneity from the analysis [53, 54]. While sensitivity analyses were conducted, there were still outcomes in the meta-analyses with an I^2^>50% which shows that large heterogeneity is present. By decreasing the heterogeneity within the sample, the relevance of the results will increase, allowing for stronger inference of conclusions [55, 56].

### Limitations

This study has a few limitations. First, we only included studies published in English. Any studies that were non-English that fit our inclusion criteria could have been overlooked. Additionally, there is some subjectivity to the Oxman & Guyatt score that was used for the methodology analysis. We conducted the score in a blinded fashion to limit the subjectivity bias and had trained Orthopaedic Surgeons to consult if we could not come to a consensus. Finally, while we used a comprehensive search strategy for the methodology, there could have been studies that were missed.

## Conclusions

This is the first overlapping analysis of overlapping systematic reviews assessing the treatment of femoral neck fractures with Cemented vs. Uncemented HAs. The best available evidence suggests that Cemented HAs are better at preventing Prosthesis-related complications. Conversely, the best evidence also suggests that Cemented HA also results in longer operative time and increased intraoperative blood loss. Both options are viable, but the physician should consider these results to determine the best avenue of treatment for each patient. In addition, until new/better research is produced to change the literature regarding Cemented vs Uncemented HAs, journals should limit the publication of systematic reviews regarding the topic.

## Supporting information

S1 Checklist PRISMA 2020 checklist. (PDF)
